# The Medicinal Halophyte *Frankenia laevis* L. (Sea Heath) Has In Vitro Antioxidant Activity, α-Glucosidase Inhibition, and Cytotoxicity towards Hepatocarcinoma Cells

**DOI:** 10.3390/plants11101353

**Published:** 2022-05-19

**Authors:** Maria João Rodrigues, József Jekő, Zoltán Cziáky, Catarina G. Pereira, Luísa Custódio

**Affiliations:** 1 Centre of Marine Sciences, Faculty of Sciences and Technology, Campus of Gambelas, University of Algarve, 8005-139 Faro, Portugal; cagpereira@ualg.pt (C.G.P.); lcustodio@ualg.pt (L.C.); 2 Agricultural and Molecular Research and Service Institute, University of Nyíregyháza, 4400 Nyíregyháza, Hungary; jeko.jozsef@nye.hu (J.J.); cziaky.zoltan@nye.hu (Z.C.)

**Keywords:** salt-tolerant plants, *Frankenia laevis*, phenolics, monoterpenes, type 2 diabetes, anti-tumor

## Abstract

This work explored the medicinal halophyte *Frankenia laevis* L. (sea heath) as a potential source of bioactive natural products. In this sense, methanol and dichloromethane extracts were prepared from aerial organs containing flowers, leaves and stems, and were profiled for their chemical composition using high-performance liquid chromatography coupled with electrospray ionization mass spectrometry (HPLC-ESI-MS/MS). The extracts were evaluated for their in vitro antioxidant capacity using five complementary methods: enzyme inhibitory effects on enzymes related with neurodegeneration (acetyl (AChE) and butyrylcholinesterase (BuChE)), Type 2 diabetes (α-glucosidase and α-amylase), hyperpigmentation/food oxidation (tyrosinase), and cytotoxicity towards human hepatocarcinoma (HepG2) cells. Fifty-one molecules were identified in the extracts, including several derivatives of phenolic acids, lignans and flavonoids, monoterpenes, and hydroxylated derivatives of linoleic acid. The methanol extract was effective in DPPH and ABTS radical scavenging (EC_50_ = 0.25 and 0.65 mg/mL, respectively), copper chelation (EC_50_ = 0.78 mg/mL), and iron reduction (EC_50_ = 0.51 mg/mL) activities, whereas the dichloromethane extract had high iron chelating ability (EC_50_ = 0.76 mg/mL). Both extracts showed the capacity to inhibit α-glucosidase, especially the dichloromethane (EC_50_ = 0.52 mg/mL). This extract also exerted a significant selective cytotoxicity towards HepG2 cells (EC_50_ = 52.1 μg/mL, SI > 1.9). In conclusion, extracts from the aerial parts of sea heath were shown to be a promising source of natural products for pharmaceutical and/or food additive applications due to their high antioxidant, anti-diabetic, and cytotoxic properties.

## 1. Introduction

Since ancient times, nature has been a source of medicines to treat different human diseases, which were at first based on empirical aspects. Nowadays, due to the beneficial properties of plant-based products, the screening of biological activities, and the identification and isolation of bioactive compounds from plants has been increasing due to the growing demand for novel compounds to provide healthcare assistance in diverse human disorders, including inflammation, cancer, diabetes, and neurological disorders [1].

The marine environment holds high biodiversity, including numerous salt-tolerant plant species called halophytes. However, the scientific and commercial interest in marine halophytes is still in its infancy, and their high biotechnological potential is almost unexplored and underutilized. These plants can thrive in environments characterized by many abiotic stresses, such as high salinity, drought, temperature variations and light intensity. Thus, in order to survive in those harsh conditions, these plants developed strong antioxidant systems that consist of antioxidant enzymes and the synthesis of protective secondary metabolites (e.g., phenolics, alkaloids, phytosterols) which also endow important therapeutic properties for humans [2].

These plants have already provided several food and herbal supplements—such as for example, quinoa seeds (*Chenopodium quinoa* Willd.), sea asparagus (*Salicornia* sp.) and sea fennel (*Crithmum maritimum* L.), which have high commercial value and are highly appreciated in gourmet cuisine [3]. While other halophyte species provide botanical extracts for health applications, namely quinoa for hair loss prevention [4]; *Hippophae rhamnoides* L. (sea buckthorn) oil for improving immunity, supporting the gastrointestinal tract, and cardiac function [5]; *Salicornia* sp. as a source of minerals and amino acids [6]; the bark of *Salvadora persica* L. as a toothpaste for oral and dental care [7]; and *Atriplex halimus* L. as a food supplement to reduce menopause symptoms [8]. These plants can thus be a promising source of bioactive metabolites with potential uses in different commercial areas (e.g., food, pharma, cosmetic), finding their place in the highly demanding markets seeking innovation, while taking advantage of their suitability to be produced using saline water resources and/or saline soils [9].

**Figure 1:**
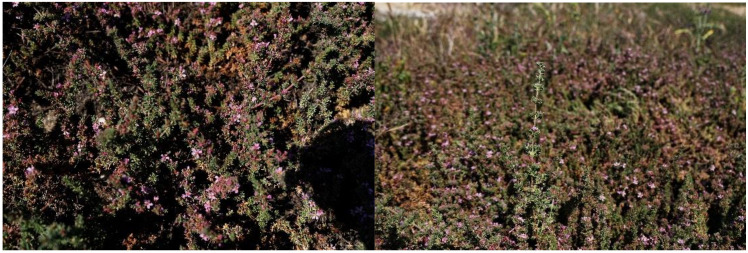
*Frankenia laevis* L. from Southern Algarve, Portugal. Photos by Chia-Yu Chu.

*Frankenia* is the most extended genus in the Frankeniaceae family, which is represented by shrubby and herbaceous species that grow in arid and semi-arid environments, growing on saline, calcareous or chalky soils [10]. It includes the species *Frankenia laevis* L. (Figure 1), commonly known as sea heath, which is distributed along the Mediterranean region and the Atlantic coast, from Portugal (including the Azores islands), Spain, and France to the west of North Africa (Algeria, Morocco, and Tunisia) [11]. 

*Frankenia* spp. is used in Asian traditional medicine in the form of an herbal tea for gargling, or for skin application due to its astringent properties, as well as in the form of tinctures to treat diverse medical conditions, such as diarrhea, dysentery, vaginal leucorrhea, gonorrhea, catarrh, and mucous problems [12]. However, there are no reports on the use of *F. laevis* in particular. In the last few decades, only two studies reported the in vitro biological potential of this species. Saïdana et al. [13] described the antibacterial activity of *F. laevis* essential oils against *Staphylococcus aureus*, *Micrococcus luteus* and *Salmonella typhimurium*, which they ascribed to the presence of hexadecenoic acid, benzyl benzoate, benzyl cinnamate, farnesyl acetate, methyl linoleate, eugenol and β-caryophyllene. Moreover, 80% aqueous acetone extracts from *F. laevis* aerial parts were described with strong in vitro radical scavenging and copper chelating activities related to their high total content in phenolic compounds [14]. Diverse sulphated phenolics were identified in whole plant aqueous–alcoholic extracts, namely sodium sulphated derivatives of acetophenone, and gallic and ellagic acids [15].

Having in mind the previously reported promising results for *F. laevis*, this work intended to further explore its biological activities and chemical composition, aiming at its valorization as a source of novel natural ingredients and/or products, with commercial applications in the food and pharmaceutical industries. For this purpose, hydrophilic (methanol) and lipophilic (dichloromethane) extracts were prepared from aboveground biomass containing flowers, leaves and stems, and were chemically characterized by high-performance liquid chromatography coupled with electrospray ionization mass spectrometry (HPLC-ESI-MS/MS). The extracts were then evaluated for in vitro radical scavenging activity (RSA) against 2,2-diphenyl-1-picrylhydrazyl (DPPH), and 2,2′-azino-bis(3-ethylbenzothiazoline-6-sulphonic acid) (ABTS), ferric reducing antioxidant power (FRAP), and copper (CCA)- and iron (ICA)-chelating activities. The inhibitory capacity of the extracts was also appraised on enzymes implicated in the onset of neurodegenerative diseases, especially Alzheimer’s disease, namely acetylcholinesterase (AChE) and butyrylcolinesterase (BuChE); hyperpigmentation disorders and food oxidation (tyrosinase); and type-2 diabetes (α-amylase and α-glucosidase). The cytotoxic potential of the extracts was evaluated towards tumoral human hepatocellular carcinoma (HepG2) cells versus the non-tumoral mouse stromal bone marrow (S17) cell line.

## 2. Results and Discussion

### 2.1. Chemical Composition

The efficiency of the extraction was evaluated by calculating the percentage of the extraction yield, with methanol being more effective than dichloromethane, presenting a yield almost 13 times higher (22% vs. 1.7%, respectively). 

The chemical profile of extracts of sea heath was established by HPLC-ESI-MS/MS, and the results are summarized in Table 1 and Figures S1–S4 (Supplementary Materials). The compounds were identified based on their accurate *m*/*z* values, retention time, and the mass spectral fragment information by comparison with data found in the literature, and when possible, with standard compounds. A total of 51 compounds were tentatively identified in the samples; there was a prevalence of hydrophilic metabolites, as the methanol extract showed higher chemical diversity, with 45 compounds detected, whereas only 20 compounds were identified in the dichloromethane extract. Besides this, several compounds were identified in both extracts, while others were only found in one of the extracts. For instance, 14 compounds were detected in both the methanol and dichloromethane extracts, namely citric acid (1), two catechol derivatives (15,20), one *O*-glycoside sulfate (16), two monoterpenes (23,24), three hydroxycinnamic acid derivatives (28,32,35), one terpene (36), two ellagic acid derivatives (38,40), one fatty acid (44), and pheophytin A (51). Conversely, six compounds were found only in the dichloromethane extracts, including some hydroxylated derivatives of unsaturated fatty acids (45,46,49,50), one precursor of jasmonic acid (47), and one long-chain fatty acid (48). The remaining 31 compounds were only extracted with methanol, namely several glycosylated and sulfated phenolic acid derivatives (2–14, 17–19,25,26,29,30,33,34,39,41–43), two sulfated lignans (21,22), and three flavonoid derivatives (27,31,37).

As is widely known, phenolics are one of the most important groups of plant secondary metabolites, which include flavonoids, phenolic acids and lignans, which are highly represented compounds in the sea heath extracts [16]. These molecules participate in many interactions between plants and the environment, such as, for example, herbivory, pigmentation, or allelopathy. Flavonoids possess several functions, namely plant development regulation, pigmentation, UV protection, and as microorganisms’ defense signaling molecules [17]. Lignans may have a defensive role as antifeedants against herbivores and microorganisms, as well as allelopathic effects [18]. The biosynthesis of these metabolites is usually increased when the plants are exposed to environmental stresses such as drought, UV radiation, and salinity, which characterize halophytes’ habitats. Moreover, enzymatic modifications result in many types of phenolic derivatives, mainly methylated, sulphated, and glycosylated compounds [19]. For example, an increased biosynthesis of sulphated phenolics is highly correlated with high salinity conditions, such as those to which halophyte plants are subjected. Despite the functional role of such compounds not being clear, it seems to be related to ecological adaptations such as co-pigmentation, growth regulation, molecular recognition, and detoxification, as the increased solubility and stability of these molecules enhance their interaction with biological targets [19]. Similarly, the accumulation of glycosylated derivatives may occur as a response to stress resistance mechanisms (e.g., high UV radiation, temperature, and salinity) working as osmoprotective, carbon storage, and free radical scavenging mechanisms [20]. Several sodium sulphated phenolics were already isolated from *F. laevis* whole-plant aqueous alcohol extract, including some gallic and ellagic acids, and acetophenone derivatives [15]. However, except for 3-O-methylgallic acid-5-O-sulfate, none of the other molecules were identified in the present work. Despite this, gallic acid has already been identified in both *F. pulverulenta* and *F. thymifolia*, linoleic acid and kaempferol were previously found in *F. thymifolia*, and some unidentified sulfated flavonoids were reported in *F. pulverulenta* [21,22]. 

Other major plant metabolites were found in *F. laevis* extracts, including citric acid, chlorophyll and jasmonic acid derivatives, which are key players in plant growth, photosynthesis, cellular respiration and photoprotection. Monoterpenes are primarily found in plant essential oils endowing allelochemical and wound-healing functions [23]. In turn, jasmonic acid is a stress-related plant hormone produced in response to environmental stresses, such as drought, salinity, and light exposure [24]. This may explain the detection of these type of metabolites in *F. laevis* extracts, as this species thrives in salt marshes subjected to severe abiotic stresses such as high salinity, drought, temperature variations, and high UV radiation. Overall, to the best of our knowledge, no previous studies have described the occurrence of most of the metabolites detected in this study in the same species or genus.

### 2.2. Biological Activities

Besides the important physiological and ecological functions of secondary plant metabolites, they are also important components for human health, contributing to the reduction of the risk of several diseases related to oxidative stress, such as inflammation, cancer, diabetes, cardiovascular disease, and neurodegeneration. In this sense, *F. laevis* extracts were further evaluated for their in vitro antioxidant activity, inhibition of enzymes related to diabetes, neurodegeneration, hyperpigmentation/food oxidation, and cytotoxic potential against human hepatocellular carcinoma cells.

#### 2.2.1. Antioxidant Activity

The extracts were evaluated for their in vitro antioxidant capacity by five complementary assays, including radical scavenging, metal chelating and iron reduction assays, and the results are presented in Table 2. Only the methanol extracts showed the ability to scavenge the DPPH and ABTS radicals, reduce iron and chelate copper more than 50% at the concentration of 1 mg/mL, allowing us to calculate the half-maximal effective concentration (EC_50_) values (0.25, 0.65, 0.51 and 0.78 mg/mL, respectively). In turn, only the dichloromethane extract was able to chelate iron, exhibiting an EC_50_ value of 0.76 mg/mL. 

As far as we know, there is only one study on the radical scavenging and metal chelation properties of *F. laevis* 80% aqueous acetone made from aboveground parts containing shoots and leaves, the obtained EC_50_ values of which were lower (DPPH: 0.12 mg/mL; ABTS: 0.18 mg/mL; CCA: 0.44 mg/mL) than those found in this work [14]; similarly to our data, the extract did not present significant ICA up to 1 mg/mL. Other species belonging to the *Frankenia* genus have already shown strong antioxidant properties, namely *F. thymifolia*, *F. triandra* and *F. pulverulenta*. In total, 80 and 96% aqueous ethanol extracts made from the aerials parts of *F. triandra* showed EC_50_ values of 37 and 15 µg/mL for ABTS radical scavenging ability and iron reducing power (FRAP), respectively [10]. Polar and non-polar fractions from *F. thymifolia* aerial organs were also described as having DPPH-radical scavenging activity and iron reducing power (EC_50_ = 99 and 120 µg/mL, correspondingly) [21]. Moreover, 80% aqueous acetone extracts from *F. pulverulenta* showed strong DPPH (EC_50_ = 0.10 mg/mL) and ABTS (EC_50_ = 0.15 mg/mL) reduction ability, as well as copper (EC_50_ = 0.50 mg/mL) and iron (EC_50_ = 0.30 mg/mL) chelation properties [14]. Likewise, ethyl acetate shoots and root fractions of *F. pulverulenta* had considerable DPPH (586 and 750 mg of TE/g) and ABTS (1453 and 1319 mg of TE/g) reducing capacity [22]. 

The significantly higher antioxidant activity of the methanol extracts can be explained by the highest amount of the compounds identified in these extracts, namely phenolic acids, lignans and flavonoid derivatives, as well as monoterpenes metabolites. Phenolic compounds are characterized by an aromatic ring carrying one or more hydroxyl groups, including derivatives, such as methyl, glycoside, or sulfate substituents. Their chemical structure is ideal for free radical scavenging due to the high capacity of hydroxyl groups to donate a hydrogen atom or an electron to a free radical, and the capacity of the conjugated aromatic system to delocalize unpaired electrons [25]. Moreover, some phenolics with dihydroxy groups, namely those containing catecholate and gallate groups, can conjugate transition metals (e.g., copper), preventing metal-induced free radical formation by the Fenton reaction [25]. Thus, the oxidation of biological molecules such as lipids, proteins and nucleic acids is reduced. In turn, monoterpenes are often linked to hydroxyl groups which confer them free radical and singlet oxygen scavenging properties, acting as effective antioxidants [26]. Instead, the iron chelation ability exhibited only by the dichloromethane extract can be linked with the presence of the unsaturated fatty acid derivatives, as iron can bind to low molecular weight biomolecules with chelating sites, including fatty acids such as linoleic acid, suggesting that their derivatives may also be able to chelate iron [27,28]. 

In addition to the antioxidant properties of the main detected metabolites (phenolic acids, lignans, flavonoids, monoterpenes, unsaturated fatty acids), they also confer important functional roles in human health and disease, exhibiting therapeutic effects against inflammation, cardiovascular, neurodegenerative diseases, cancer, diabetes, and obesity problems [29,30,31,32]. Thus, natural sources rich in these compounds, such as *F. laevis*, have the potential to be used to provide antioxidant supplements by free radicals’ inactivation and/or metal chelation, leading to reduced cellular damage and associated disease development. Moreover, such compounds also have applications in the food industry to prevent food oxidation, which leads to the loss of important nutritional and sensory food properties [33]. In fact, the interest in using natural antioxidants to replace their synthetic counterparts, such as BHT (E320), is rising due to many factors, including the undesirable side effects of the latter ones, which include carcinogenic properties, and the additional health benefits displayed by the former [33]. 

#### 2.2.2. Enzyme Inhibition

One of the most common therapeutic tools to manage human diseases is the inhibition of key enzymes associated with those problems, contributing to symptom relief [34]. In this sense, the inhibitory capacity of *F. laevis* extracts was therefore evaluated on enzymes implicated in the onset of neurodegenerative diseases, especially Alzheimer’s disease (AChE and BuChE), hyperpigmentation disorders (tyrosinase) and diabetes (α-amylase and α-glucosidase) (Table 3). The most common prophylactic treatment for type 2 diabetes includes reducing carbohydrate digestibility by inhibiting two key hydrolyzing enzymes, namely α-amylase and α-glucosidase, to reduce postprandial hyperglycemia. However, the secondary effects presented by clinically used inhibitors (e.g., acarbose, miglitol, voglibose), which may include diarrhea and abdominal pain, emphasize the demand to search for novel natural therapeutic compounds with fewer harmful effects [35]. Both the methanol and dichloromethane extracts of *F. laevis* had the ability to inhibit the α-glucosidase enzyme, but the latter exhibited the lowest EC_50_ value (0.52 mg/mL), which was six-times inferior to that of the positive control, acarbose (EC_50_ = 3.14 mg/mL). To the best of our knowledge, there were no previous studies reporting the α-glucosidase inhibitory properties of the *Frankenia* genus. 

Many bioactive compounds, such as phenolic compounds (e.g., flavonoids, tannins, lignans), may be safer alternatives to primary pharmacological therapies, and may also contribute to the reduction of the occurrence of secondary diabetic complications linked to oxidative stress [35]. For instance, linoleic acid and its derivatives, such as those detected in *F. laevis* dichloromethane extract, are reported to be competitive inhibitors of α-glucosidase, and are more potent than the positive control, acarbose, and with weaker anti-α-amylase activity, which was similar to that observed in our work [36]. Loliolide, a monoterpene present in both *F. laevis* methanol and dichloromethane extracts, is described to have a strong inhibitory effect against α-glucosidase, with EC_50_ values of 388.48 µM (approx. 76 µg/mL); thus, its presence, as well as that of the structurally related molecules of isololiolide and dihydroactinidiolide, may explain the anti-α-glucosidase activity of *F. laevis* extracts. Furthermore, the higher abundance of these compounds in the dichloromethane extract may be related to its higher α-glucosidase inhibition [37]. Despite the lower EC_50_ value of the methanol extract, its α-glucosidase inhibitory activity could be due to the presence of several molecules previously reported with the capacity to inhibit carbohydrate-hydrolyzing enzymes, namely phenolic acids (e.g., gallic, ferulic, coumaric and caffeic acids) [38], lignans (e.g., lariciresinol) [39], and flavonoids (e.g., kaempferol) [40]. Overall, the *F. laevis* extracts were shown to hold the potential to be used as a source of α-glucosidase inhibitors, contributing to the reduction of postprandial hyperglycemia in type 2 diabetes patients. Moreover, the antioxidant ability of these extracts may also contribute to the reduction of the risk of diabetic complications associated with oxidative stress, namely microvascular and cardiovascular complications [41,42]. 

Conversely, *F. laevis* extracts did not demonstrate the ability to inhibit any of the other enzymes tested, namely AChE, BuChE, α-amylase and tyrosinase. However, methanol extracts from this species have previously shown high AChE and BuChE inhibition (approx. 80% at 1 mg/mL) [43]. In turn, similarly to our work, 80% aqueous acetone extracts of *F. laevis* aerial organs also did not show any capacity to inhibit tyrosinase at the concentration of 1 mg/mL [14]. 

#### 2.2.3. Cytotoxicity

Hepatocellular carcinoma is one of the main liver tumors that are generally derived from chronic liver diseases, such as cirrhosis or hepatitis. It is an aggressive cancer with high metastatic capability and high resistance to cytotoxic drugs, which leads to the need to identify new drug leads for hepatocarcinoma chemotherapeutics [44]. In this respect, the cytotoxic potential of *F. laevis* extracts was assessed on tumoral human hepatocellular carcinoma (HepG2) cells in comparison with the non-tumoral mouse stromal bone marrow (S17) cell line. The results are presented in Table 4. 

A significant reduction in HepG2 cell viability was observed after the application of the dichloromethane extract of *F. laevis* with an EC_50_ value of 52.1 µg/mL. Conversely, this extract did not show significant cytotoxicity towards the non-tumoral S17 cell line up to the maximum concentration tested (100 µg/mL); as such, it exhibited a selectivity index (SI) of at least 1.9 (Table 4), which means that the sample is less toxic for normal cells than tumoral ones, and that it is safer for therapeutic uses [45]. The methanol extract also did not show significant cytotoxicity up to 100 µg/mL on both HepG2 and S17 cells (Table 4). 

**Figure 2:**
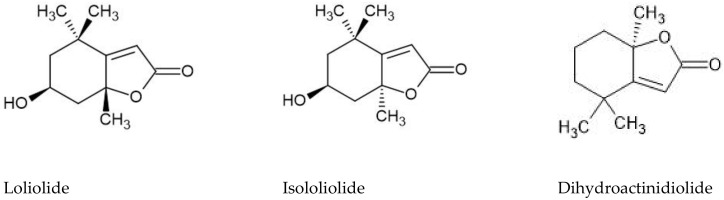
Chemical structures of loliolide, isololiolide and dihydroactinidiolide.

To the best of our knowledge, this is the first report on the in vitro anti-hepatocarcinoma potential of *Frankenia* species. However, the monoterpene metabolites detected in higher abundance in the dichloromethane extract from *F. laevis* aerial parts, such as isololiolide and loliolide (Figure 2), have already been isolated from marine macro- and microalgae, respectively, and were ascribed with selective cytotoxicity against HepG2 cells [46,47]. 

Dihydroactinidiolide (Figure 2), a structural analog of loliolide, also showed a strong cytotoxic effect on human lung carcinoma cells (A549) [48], as well as an enriched fraction of *Moringa stenopetala* in loliolide and dihydroactinidiolide that was highly cytotoxic against human hepatocarcinoma (HepG2) and human breast adenocarcinoma (MCF-7) cells, with EC_50_ values ranging between 35 and 39 µg/mL [49]. Furthermore, the polyunsaturated fatty acid linoleic acid has been described to reduce the generation of pre-cancerous hepatic nodules in rats, demonstrating its potential chemoprotective effect on hepatocellular carcinoma [50,51]. Oxophytodienoic acid, a phytohormone with plant growth and development functions, was also described to inhibit human breast cancer cells’ proliferation by decreasing the expression of cyclin D1 [52]. Thus, the anti-hepatocarcinoma activity of *F. laevis* dichloromethane extract can also be ascribed to linoleic acid’s hydroxylated derivatives and the oxophytodienoic acid found in this extract. According to this, *F. laevis* dichloromethane extracts were shown to be a promising source of molecules with possible applications as a selective anti-hepatocarcinoma therapy, being a candidate for further studies on the identification and isolation of antitumoral lead drugs.

## 3. Materials and Methods

### 3.1. Chemicals

The DPPH and ABTS radicals, BHT, AChE from electric eels (EC 3.1.1.7), equine BuChE (EC 3.1.1.8), acetylthiocholine iodide, butyrylthiocholine iodide, 5-thio-2-nitrobenzoate (DTNB), tyrosinase from mushrooms (EC 1.14.18.1), L-tyrosine, α-amylase from porcine pancreas (EC 3.2.1.1), and α-glucosidase from *Saccharomyces cerevisiae* (EC 3.2.1.20) were purchased from Sigma-Aldrich (Lisbon, Portugal). Additional reagents and solvents were obtained from VWR International (Leuven, Belgium). 

### 3.2. Plant Material 

The aerial organs of *F. laevis* at the flowering stage, including stems, leaves, and flowers at anthesis and prior to anthesis, were collected in the southwest of Portugal (Algarve) (coordinates: 43°38′19.39″ N 116°14′28.86″ W) in June of 2019. The taxonomical classification was determined by the botanist Dr. Manuel J. Pinto (National Museum of Natural History, University of Lisbon, Botanical Garden, Portugal). The samples were oven dried for 3 days at 50 °C, powdered, and stored at −20 °C until needed.

### 3.3. Extraction

Dried samples were separately mixed with methanol and dichloromethane (1:40 *w*/*v*) and were extracted overnight at room temperature (RT), under stirring. The extracts were filtered (Whatman n° 4) and evaporated under a vacuum. The dried extracts were dissolved in dimethyl sulfoxide (DMSO) at the concentration of 10 mg/mL, and were stored at −20 °C. 

### 3.4. High-Performance Liquid Chromatography Coupled with Electrospray Ionization Mass Spectrometry (Hplc-Esi-MS/Ms)

The chemical composition of the extracts was determined using a Dionex Ultimate 3000RS UHPLC instrument. The extracts were filtered through a 0.22 μm PTFE filter membrane (Labex Ltd., Hungary) before HPLC analysis. The extracts were injected onto a Thermo Accucore C18 (100 mm × 2.1 mm, i. d., 2.6 μm) column thermostated at 25 °C (± 1 °C). The solvents used were water (A) and methanol (B), both acidified with 0.1% formic acid. The flow rate was maintained at 0.2 mL/min. The elution gradient was isocratic 5% B (0–3 min), a linear gradient increasing from 5% B to 100% (3–43 min), 100% B (43–61 min), a linear gradient decreasing from 100% B to 5% (61–62 min), and 5% B (62−70 min). The column was coupled with a Thermo Q-Exactive Orbitrap mass spectrometer (Thermo Scientific, Waltham, MA, USA) equipped with an electrospray ionization source. The spectra were recorded in positive- and negative-ion mode, respectively. The trace finder 3.1 (Thermo Scientific, Waltham, MA, USA) software was applied for target screening. Most of the compounds were identified based on our previously published work or data found in the literature. In every case, the exact molecular mass, isotopic pattern, characteristic fragment ions and retention time were used for the identification of the compounds which are marked and were confirmed by standards. The difference between the measured and calculated molecular mass was less than 5 ppm in each case.

### 3.5. Determination of the In Vitro Biological Activities 

The samples’ activities were determined at 1 mg/mL, and when the activity was higher than 50%, different concentrations (0.03125, 0.0625, 0.125, 0.25, 0.5, and 1 mg/mL) were tested for the determination of the EC_50_ values (mg/mL). The absorbance was measured in a microplate reader (Biotek Synergy 4), and the activity was calculated as a percentage of inhibition, relative to a control containing DMSO in place of the sample.

#### 3.5.1. Determination of the Antioxidant Activity

##### RSA on DPPH and ABTS Radicals

The extracts were tested on DPPH and ABTS, as described elsewhere [53]. The samples were mixed with radical solutions (DPPH: 120 µM; ABTS: 7.4 mM) in 96-well flat-bottom microtitration plates and were incubated in darkness at RT for 30 and 6 min, respectively. The synthetic antioxidant BHT (E320), used as a preservative in food and cosmetics, was used as a positive control at the same concentrations of the samples. The absorbance was measured at 517 and 734 nm for DPPH and ABTS, respectively.

##### FRAP

The ability of the extracts to reduce Fe^3+^ was evaluated as previously described by Rodrigues et al. [53]. The samples were mixed with distilled water and 1% potassium ferricyanide in 96-well plates, and were incubated at 50 °C for 20 min. Then, 10% trichloroacetic acid and 0.1% ferric chloride solution were added. Increased absorbance at 700 nm means higher reducing activity, and the results were expressed as a percentage of inhibition relative to the positive control at the concentration of 1 mg/mL.

##### CCA and ICA

The CCA and ICA of the extracts and the positive control (EDTA) were assessed in 96-well microplates according to the methods described in Rodrigues et al. [53]. For CCA, the samples were mixed with 50 mM Na acetate buffer (pH 6), 4 mM pyrocatechol violet, and 50 µg/mL CuSO_4_ solution. For ICA, the samples were mixed with distilled water and 0.1 mg/mL FeCl_2_ solution. After 30 min, a 40 mM ferrozine solution was added. The change in absorbance was measured at 632 and 562 nm for CCA and ICA, correspondingly. 

#### 3.5.2. Determination of the Enzyme Inhibitory Activity

##### AChE and BuChE Inhibitory Activities

The inhibitory effect of the extracts and the standard (galantamine) on AChE and BuChE was assessed according to the method explained by Custódio et al. [54]. The samples were mixed with 0.02 M phosphate buffer (pH 8.0) and enzyme solution (0.28 U/mL). After 15 min at 25 °C, the substrate (acetylcholine iodide or butyrylcholine chloride, 4 mg/mL) and 5,5′-dithiobis-(2-nitrobenzoic acid) (DTNB, 1.2 mg/mL) were added and incubated for 15 min at 25 °C. The changes in the absorbances were measured at 412 nm.

##### Tyrosinase Inhibitory Activity

The tyrosinase inhibitory activity of the extracts and the positive control, arbutin, a commercially available tyrosinase inhibitor, were assessed as reported before [55]. The samples were mixed with enzyme solution (333 U/mL) in 25 mM potassium phosphate buffer (pH 6.5). After 5 min, the substrate L-tyrosine (2 mM) was added and incubated for an additional 30 min. period, at room temperature. The optical densities were read at 492 nm. 

##### α-Amylase and α-Glucosidase Inhibitory Activities

The α-amylase and α-glucosidase inhibitory activities of the extracts and of the positive control, acarbose, the active ingredient of a clinically used drug for the control of type 2 diabetes (Glucobay^®^), were determined by the method depicted in Rodrigues et al. [56]. For α-amylase, the samples were mixed with amylase solution (100 U/mL) and 0.1% starch solution. After 10 min at 37 °C, 1 M hydrochloric acid (HCl) and 5 mM iodide solution were added. For α-glucosidase, the extracts were mixed with enzyme solution (1.0 U/mL) and incubated for 10 min. at 25 °C. Then, 5 mM of substrate solution (*p*-nitrophenyl-α-*D*-glucopyranoside) was added and incubated for a further 5 min at 25 °C. The absorbance was measured at 580 and 405 nm for α-amylase and α-glucosidase, respectively. 

#### 3.5.3. Determination of the Cytotoxic Activity

##### Cell Culture 

The HepG2 (human hepatocellular carcinoma) and S17 (murine bone marrow stromal) cell lines were maintained in Dulbecco’s modified eagle medium (DMEM) culture medium supplemented with 10% heat-inactivated fetal bovine serum (FBS), 1% L-glutamine (2 mM), and 1% penicillin (50 U/mL)/streptomycin (50 μg/mL) and were maintained at 37 °C in humidified atmosphere with 5% CO_2_.

##### Cellular Viability Assay

HepG2 and S17 cells were plated in 96-well tissue plates at a density of 5 × 10^3^ cells/well and were incubated for 24 h. Then, the extracts were applied at several concentrations (3.125, 6.25, 12.5, 25, 50 and 100 µg/mL) for 72 h. The 3-(4,5-dimethylthiazol-2-yl)-2,5-diphenyltetrazolium bromide (MTT) colorimetric test was used to determine the cellular viability (Biotek Synergy 4), as formerly detailed [57]. The absorbance was measured at 590 nm, and the results were expressed in terms of cellular viability (%) in relation to a control containing DMSO (0.5%) and EC_50_ values (µg/mL). The selectivity index (SI) was obtained by dividing the EC_50_ value of non-tumoral cells (S17) by the EC_50_ of tumoral cells (HepG2).

### 3.6. Statistical Analysis

The results were expressed as the mean ± standard error of the mean (SEM), and the experiments were conducted at least in triplicate. Significant differences were assessed by Student’s *t*-test. *p* values lower than 0.05 were considered significant. All the statistical analysis was performed using the XLSTAT statistical package for Microsoft Excel (version 2013, Microsoft Corporation). The IC_50_ values were calculated by the sigmoidal fitting of the data using GraphPad Prism version 9.

## 4. Conclusions

This work reported the in vitro antioxidant activity, enzyme inhibition and cytotoxicity towards hepatocellular carcinoma cells of extracts made from the aerial organs of the medicinal halophyte *F. laevis*. The methanol extract had a high antioxidant activity against DPPH and ABTS radicals, high copper chelating ability, and iron reducing power, whilst the dichloromethane extract showed high iron chelation capacity. Both extracts had high inhibitory activity towards the α-glucosidase enzyme, and the dichloromethane extract exerted a selective cytotoxic effect on the human hepatocellular carcinoma (HepG2) cell line. However, the sea heath extracts did not display significant AChE and BuChE, α-amylase or tyrosinase inhibition. The chemical profiling of the sea heath extracts detected several derivatives of phenolic acids, lignans and flavonoids, monoterpenes, and hydroxylated derivatives of linoleic acid. These molecules have been reported to have biological activities, namely antioxidant, antidiabetic, and anti-tumor activities, which suggest that the aerial parts of *F. laevis* are a promising source of molecules with potential applications in pharmaceutical and/or food industries, as new drug leads, herbal health supplements, and/or food preservatives.

## Figures and Tables

**Table 1:** HPLC-ESI-MS/MS tentative identification of the metabolites present in the methanol and dichloromethane extracts of the aerial parts of sea heath (*F. laevis*).

No.	Name	Formula	Rt	[M + H]^+^	[M − H]^−^	Methanol	Dichloromethane
1 ^1^	Citric acid	C_6_H_8_O_7_	2.57		191.01918	++	+
2 ^1^	Gallic acid (3,4,5-Trihydroxybenzoic acid)	C_7_H_6_O_5_	3.25		169.01370	+	-
3	Gallic acid sulfate	C_7_H_6_O_8_S	3.55		248.97051	+	-
4 ^2^	3-*O*-Methylgallic acid-5-*O*-sulfate	C_8_H_8_O_8_S	8.69		262.98616	+	-
5	Uralenneoside	C_12_H_14_O_8_	11.09		285.06105	+	-
6	(Trihydroxyphenyl)propanoic acid hexoside-*O*-sulfate	C_15_H_20_O_13_S	11.80		439.05464	+	-
7	Benzylhexose sulfate	C_13_H_18_O_9_S	11.95		349.05933	+	-
8	Dihydroxy-methoxybenzoic acid	C_8_H_8_O_5_	12.13		183.02935	+	-
9	Feruloylhexose sulfate	C_16_H_20_O_12_S	12.50		435.05972	+	-
10	Caffeoylhexose sulfate	C_15_H_18_O_12_S	12.79		421.04408	+	-
11	Caffeic acid sulfate	C_9_H_8_O_7_S	13.89		258.99125	+	-
12	Coumaroylhexose sulfate	C_15_H_18_O_11_S	13.92		405.04916	+	-
13	*p*-Coumaric acid 4-*O*-sulfate	C_9_H_8_O_6_S	14.26		242.99634	+	-
14	Unidentified sulfate (Vanillin derivative)	C_20_H_22_O_9_S	15.11		437.09063	+	-
15	Butenylpyrocatechol sulfate or isomer	C_10_H_12_O_5_S	15.54		243.03272	++	+
16	Phenylethylhexose sulfate	C_14_H_20_O_9_S	15.71		363.07498	++	+
17	12-Hydroxyjasmonic acid sulfate or Tuberonic acid sulfate	C_12_H_18_O_7_S	16.19		305.06950	+	-
18	Caffeoylpentose sulfate	C_14_H_16_O_11_S	16.40		391.03351	+	-
19	Ferulic acid 4-*O*-sulfate	C_10_H_10_O_7_S	16.51		273.00690	+	-
20	Butanoylpyrocatechol sulfate or isomer	C_10_H_14_O_5_S	16.69		245.04837	++	+
21	Lyoniresinol sulfate	C_22_H_28_O_11_S	17.59		499.12741	+	-
22	Lariciresinol or isomer sulfate	C_20_H_24_O_9_S	18.21		439.10628	+	-
23	Isololiolide	C_11_H_16_O_3_	18.54	197.11777		+	++
24	Loliolide	C_11_H_16_O_3_	19.75	197.11777		+	++
25	3-*O*-Methylellagic acid-4′-*O*-glucoside	C_21_H_18_O_13_	21.30		477.06692	+	-
26	3,3′-Di-*O*-methylellagic acid-4-*O*-glucoside	C_22_H_20_O_13_	21.76		491.08257	+	-
27	Isorhamnetin-*O*-pentosylhexoside	C_27_H_30_O_16_	21.99		609.14556	+	-
28	*N*-*cis*-Feruloyltyramine	C_18_H_19_NO_4_	22.28	314.13924		+	++
29	Ellagic acid	C_14_H_6_O_8_	22.79		300.99845	+	-
30	3-*O*-Methylellagic acid-4-*O*-sulfate	C_15_H_8_O_11_S	24.10		394.97091	+	-
31	Kaempferol sulfate isomer 1	C_15_H_10_O_9_S	24.53		364.99673	+	-
32	N-*trans*-Feruloyltyramine	C_18_H_19_NO_4_	24.55	314.13924		+	++
33	3,3′-Di-*O*-methylellagic acid-4-O-sulfate	C_16_H_10_O_11_S	24.82		408.98656	+	-
34	3-*O*-Methylellagic acid	C_15_H_8_O_8_	25.19		315.01410	+	-
35	*N*1,*N*5,*N*10-Tricoumaroylspermidine isomer 1	C_34_H_37_N_3_O_6_	26.22		582.26042	++	+
36	Dihydroactinidiolide	C_11_H_16_O_2_	26.58	181.12286		+	++
37	Kaempferol sulfate isomer 2	C_15_H_10_O_9_S	26.60		364.99673	+	-
38	3,3′,4-Tri-*O*-methylellagic acid-4′-*O*-sulfate	C_17_H_12_O_11_S	26.99		423.00221	++	+
39	*N*1,*N*5,*N*10-Tricoumaroylspermidine isomer 2	C_34_H_37_N_3_O_6_	27.25		582.26042	+	-
40	3,3′-Di-*O*-methylellagic acid	C_16_H_10_O_8_	27.45		329.02975	+	++
41	*N*1,*N*5,*N*10-Tricoumaroylspermidine isomer 3	C_34_H_37_N_3_O_6_	28.10		582.26042	+	-
42	*N*1,*N*5,*N*10-Tricoumaroylspermidine isomer 4	C_34_H_37_N_3_O_6_	28.86		582.26042	+	-
43	3,3′,4-Tri-*O*-methylellagic acid	C_17_H_12_O_8_	29.90		343.04540	+	-
44	Malyngic acid	C_18_H_32_O_5_	32.28		327.21715	+	++
45	Hydroxyoctadecatrienoic acid isomer 1	C_18_H_30_O_3_	39.73		293.21167	-	+
46	Hydroxyoctadecatrienoic acid isomer 2	C_18_H_30_O_3_	39.95		293.21167	-	+
47	Oxophytodienoic acid	C_18_H_28_O_3_	40.00		291.19603	-	+
48	Hexadecanedioic acid	C_16_H_30_O_4_	40.53		285.20659	-	+
49	Hydroxyoctadecatrienoic acid isomer 3	C_18_H_30_O_3_	41.11		293.21167	-	+
50	Hydroxyoctadecadienoic acid	C_18_H_32_O_3_	41.16		295.22732	-	+
51	Pheophytin A	C_55_H_74_N_4_O_5_	62.57	871.57375		+	++

HPLC-ESI-MS/MS: high-performance liquid chromatography coupled with electrospray ionization mass spectrometry. Rt: retention time. +: Presence; ++: Presence in higher abundance; -: Absence. ^1^ Confirmed by standard. ^2^ Hussein [15].

**Table 2:** Antioxidant activities of the methanol and dichloromethane extracts of sea heath’s (*F. laevis*) aboveground biomass. The results are expressed as half-maximal effective concentration (EC_50_) values (mg/mL).

Assay	Methanol	Dichloromethane	Positive Control
RSA-DPPH	0.25 ± 0.01 ^b^	>1 mg/mL	0.11 ± 0.00 ^a^
RSA-ABTS	0.65 ± 0.02 ^b^	>1 mg/mL	0.06 ± 0.00 ^a^
FRAP	0.51 ± 0.03	>1 mg/mL	na
CCA	0.78 ± 0.01 ^b^	>1 mg/mL	0.17 ± 0.00 ^a^
ICA	>1 mg/mL	0.76 ± 0.05 ^b^	0.06 ± 0.00 ^a^

Values represent the mean ± standard error of the mean (SEM) of at least three experiments performed in triplicate (*n* = 9). In the same line, the values followed by different letters (a, b) are significantly different at *p* < 0.05 (Student’s *t*-test). RSA–DPPH: radical scavenging activity on 2,2-diphenyl-1-picrylhydrazyl; RSA–ABTS: radical scavenging activity on 2,2′-azino-bis(3-ethylbenzothiazoline-6-sulphonic acid; FRAP: ferric reducing antioxidant power; CCA: copper chelating activity; ICA: iron chelating activity; na: not applicable. Positive controls: butylated hydroxytoluene (BHT, E320) for DPPH, ABTS and FRAP, and ethylenediamine tetraacetic acid (EDTA) for CCA and ICA.

**Table 3:** Enzyme inhibitory activities of the methanol and dichloromethane extracts of the aerial parts of sea heath (*F. laevis*). The results are expressed as half-maximal effective concentration (EC_50_) values (mg/mL).

Enzyme	Methanol	Dichloromethane	Positive Control
α-glucosidase	1.02 ± 0.01 ^b^	0.52 ± 0.04 ^a^	3.14 ± 0.23 ^c^
α-amylase	>1 mg/mL	>1 mg/mL	7.80 ± 0.17
AChE	>1 mg/mL	>1 mg/mL	0.01 ± 0.00
BuChE	>1 mg/mL	>1 mg/mL	0.32 ± 0.01
Tyrosinase	>1 mg/mL	>1 mg/mL	0.17 ± 0.01

Values represent the mean ± standard error of the mean (SEM) of at least three experiments, each performed in triplicate (*n* = 9). In the same line, values followed by different letters (a–c) are significantly different at *p* < 0.05 (student’s *t*-test). AChE: acetylcholinesterase; BuChE: butyrylcolinesterase. Positive controls: acarbose (α-amylase and α-glucosidase), galantamine (AChE and BuChE), and arbutin (tyrosinase).

**Table 4:** Cytotoxic activity of methanol and dichloromethane extracts of the aerial parts of sea heath (*F. laevis*) towards human hepatocarcinoma (HepG2) and mouse bone marrow stromal (S17) cell lines. The results are expressed as half-maximal effective concentration (EC_50_) values (µg/mL).

Cell Line	Methanol	Dichloromethane	Positive Control
HepG2	>100 µg/mL	52.1 ± 2.5 ^b^ (SI > 1.9)	1.45 ± 0.15 ^a^
S17	>100 µg/mL	>100 µg/mL	7.86 ± 0.25

Values represent the mean ± standard error of the mean (SEM) of at least three experiments, each performed in triplicate (*n* = 9). In the same line, values followed by different letters (a, b) are significantly different at *p* < 0.05 (Student’s *t*-test). SI: selectivity index. Positive control: etoposide.

## Data Availability

The dataset is available upon request from the corresponding author.

## Supplementary Materials

The following are available online at https://www.mdpi.com/article/10.3390/plants11101353/s1, Figure S1: Total ion chromatogram (positive mode) of *F. laevis* methanol extract. Figure S2: Total ion chromatogram (negative mode) of *F. laevis* methanol extract. Figure S3: Total ion chromatogram (positive mode) of *F. laevis* dichloromethane extract. Figure S4: Total ion chromatogram (negative mode) of *F. laevis* dichloromethane extract.
