# Supplementary material for: The Medicinal Halophyte Frankenia laevis L. (Sea Heath) Has In Vitro Antioxidant Activity, α-Glucosidase Inhibition, and Cytotoxicity towards Hepatocarcinoma Cells
Source: Plants (Basel). 2022 May 19;11(10):1353. doi: 10.3390/plants11101353 (PMC9148066; doi:10.3390/plants11101353)
Supplement: Supplementary file 1 [file plants-11-01353-s001.zip › plants-1723989-supplementary.pdf]

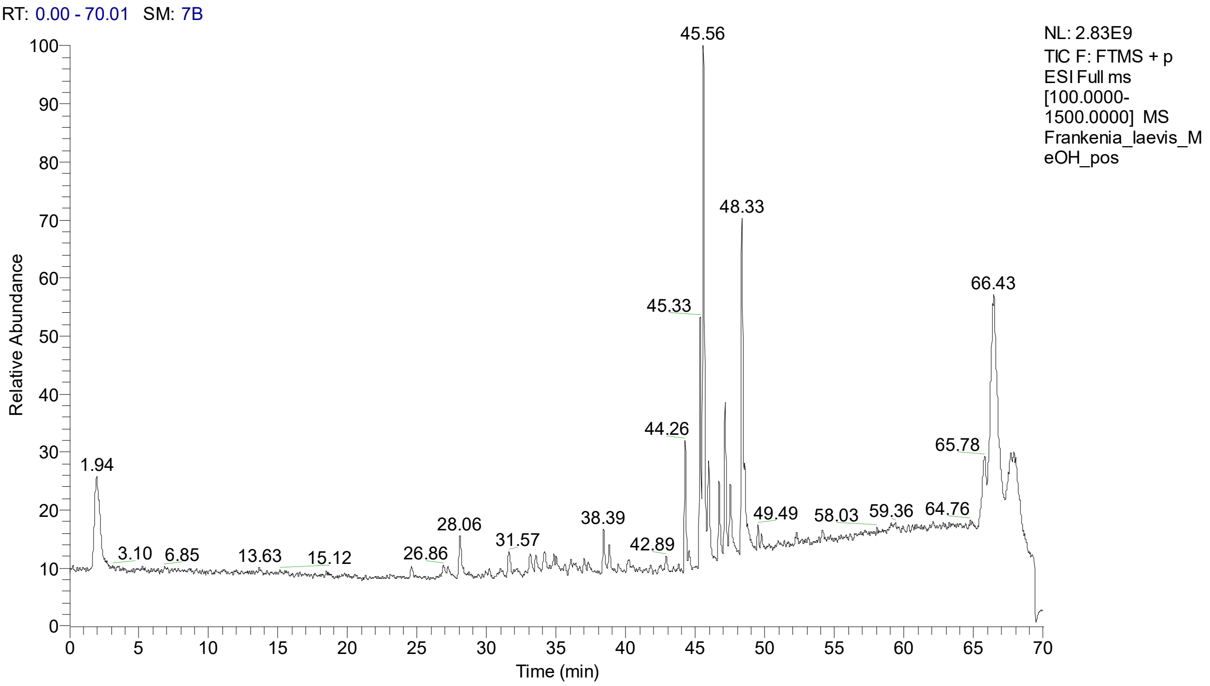

Figure S1. Total ion chromatogram (positive mode) of *F. laevis* methanol extract.

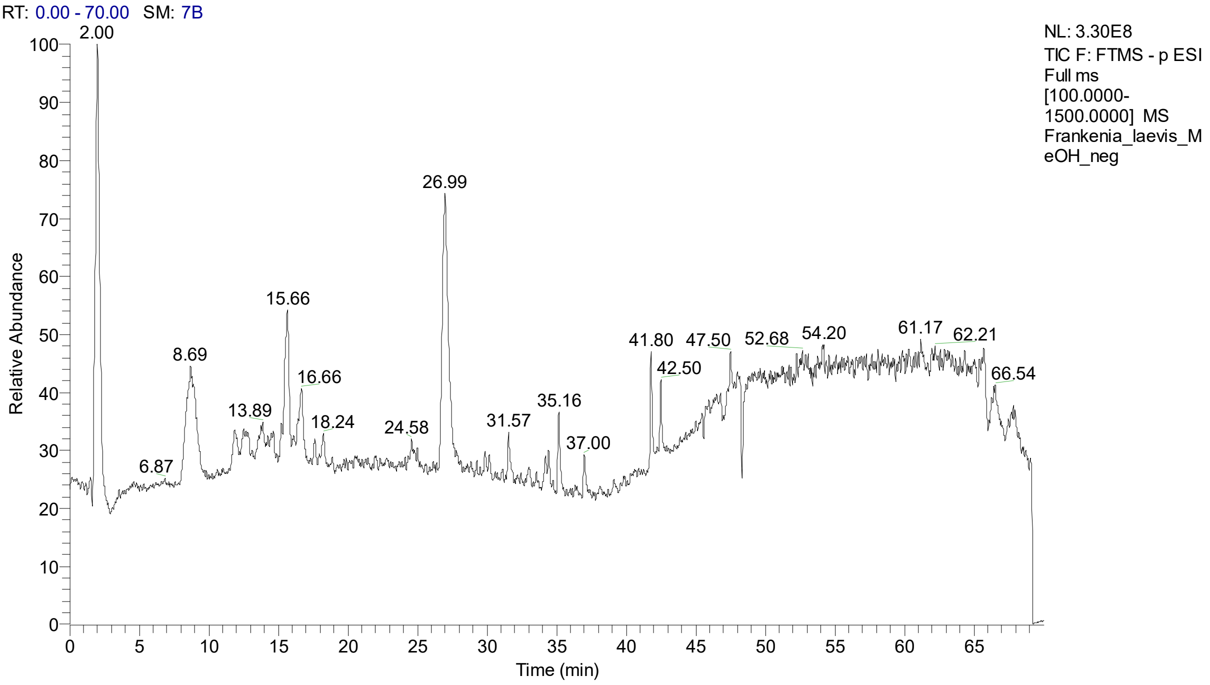

Figure S2. Total ion chromatogram (negative mode) of *F. laevis* methanol extract.

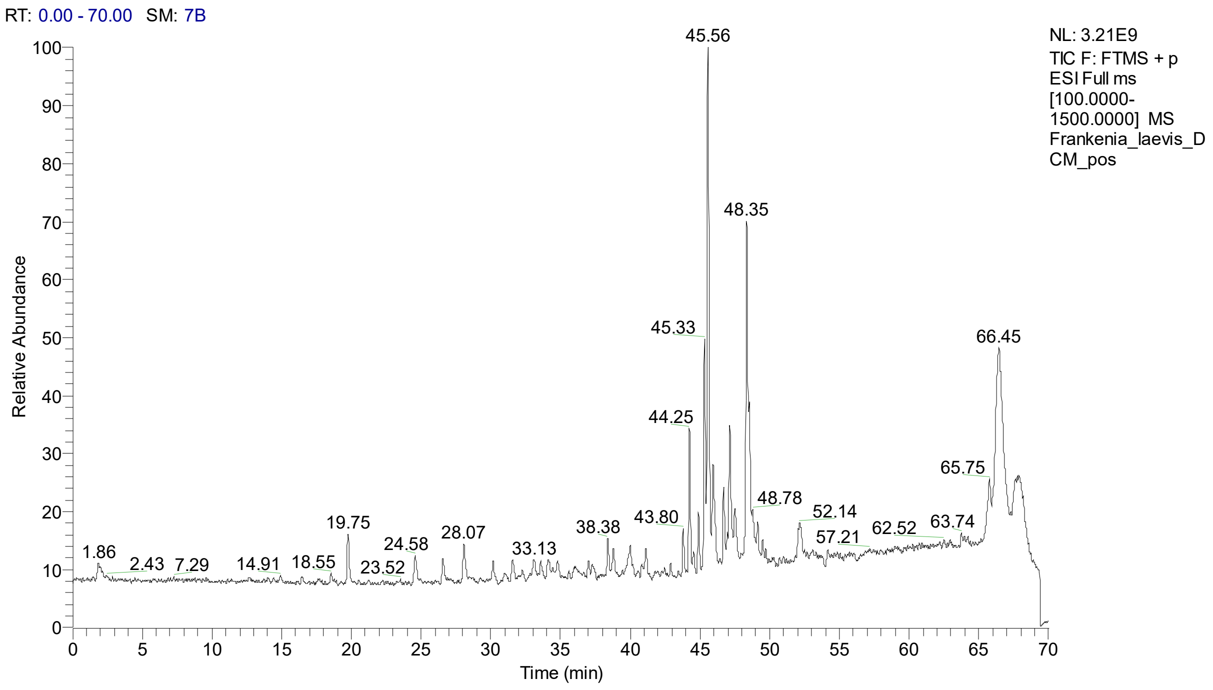

Figure S3. Total ion chromatogram (positive mode) of *F. laevis* dichloromethane extract.

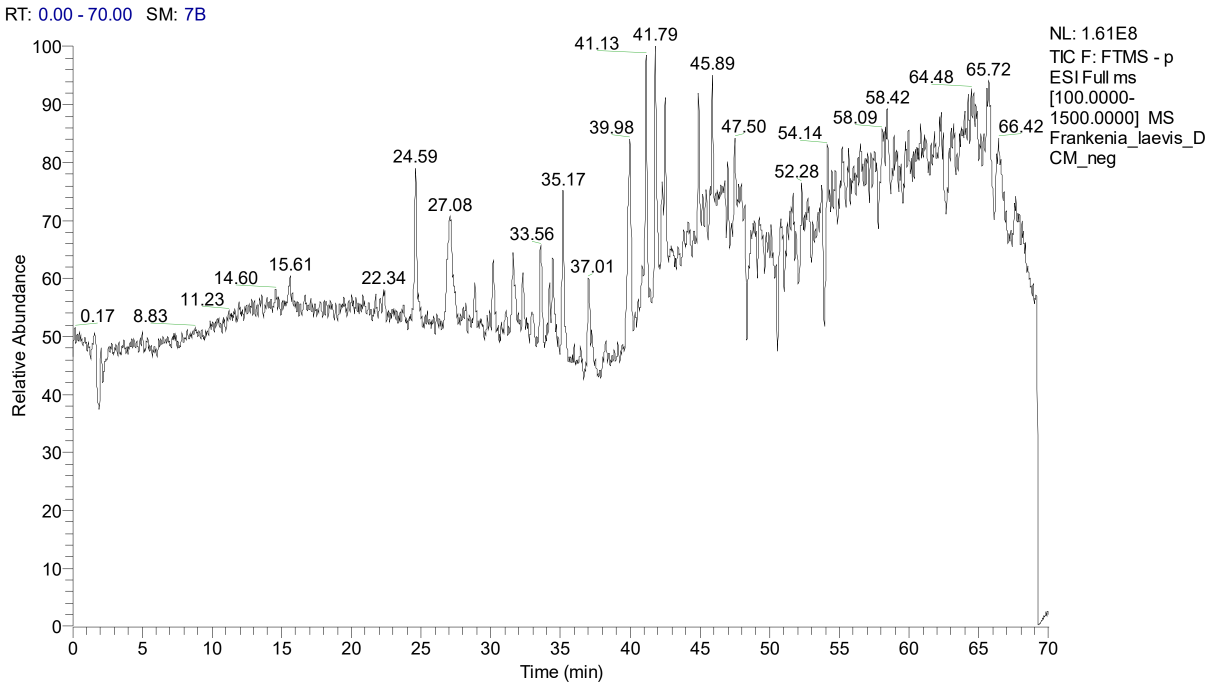

Figure S4. Total ion chromatogram (negative mode) of *F. laevis* dichloromethane extract.
